# Delusional parasitosis secondary to vascular pathology in an oncological patient: case study

**DOI:** 10.1192/j.eurpsy.2025.1772

**Published:** 2025-08-26

**Authors:** M. Garza, A. Alcorta, C. Martinez, A. Cantú, S. Reyes

**Affiliations:** 1 Psicooncología, Medicina de Enlace y Cuidados Paliativos; 2 Psychiatry, Hospital Universitario Dr José Eleuterio González, Monterrey, Mexico

## Abstract

**Introduction:**

Delusional parasitosis is a psychotic disorder where individuals firmly believe they are infested with parasites despite no medical evidence. It can be primary or secondary, the latter being a symptom of other medical conditions like neurological diseases. Effective management requires collaboration between psychiatry and other specialties.

**Objectives:**

The evaluation of each patient with an interdisciplinary team increases adherence to treatment in patients with cancer and psychiatric illness.

**Methods:**

Clinical history, complementary studies, and review of the literature on the case of a 66-year-old woman subjected to multiple dermatological treatments due to a sensation of body infestation. History of type 2 diabetes and diabetic neuropathy since 2021. In 2022, she was diagnosed with stage IIIA luminal B breast cancer and treated with surgery, chemotherapy, and radiotherapy. She is currently on adjuvant treatment with Anastrozole. The oncologist referred her due to the presence of psychotic symptoms. The General Health Questionnaire (GHQ-28), Functional Assessment of Cancer Therapy—General (FACT-G), and Positive and Negative Syndrome Scale (PANSS) were applied.

**Results:**

The patient presents with a psychotic disorder secondary to vascular pathology, manifested by delusions of infestation and sudden-onset hypodermic tactile hallucinations. Test results show a GHQ-28 score of 10/84, FACT score of 24/108, and PANSS score of 49 points. Although denying affective symptoms, anxiety, or cognitive impairment, neurological findings indicate decreased brain parenchyma, suggesting small vessel disease. Treatment includes Risperidone 1 mg once daily, along with therapeutic interventions such as psychoeducation and continued multidisciplinary monitoring by neurology for comprehensive disease management.

**Conclusions:**

Evaluating psychotic symptoms requires assessing organic and non-organic factors. Neuroimaging aids diagnosing delusional parasitosis, improving treatment through interdisciplinary collaboration.

**Disclosure of Interest:**

None Declared

